# Efficacy comparison of transcervical video-assisted mediastinoscopic lymphadenectomy combined with left transthoracic esophagectomy versus right transthoracic esophagectomy for esophageal cancer treatment

**DOI:** 10.1186/s12957-017-1268-3

**Published:** 2018-02-09

**Authors:** Xu Li, Wenxiang Wang, Yong Zhou, Desong Yang, Jie Wu, Baihua Zhang, Zhining Wu, Jinming Tang

**Affiliations:** 1 Department of Thoracic Surgery, The Second Xiangya Hospital of Central South University, Changsha, Hunan 410011 China; 2 The Second Department of Thoracic Surgery, Hunan Cancer Hospital and The Affiliated Cancer Hospital of Xiangya School of Medicine, Central South University, No.283 Tongzipo Street, Yuelu District, Changsha, Hunan 410013 China

**Keywords:** Esophageal carcinoma, Esophagectomy, Recurrent laryngeal nerve, Video-assisted mediastinoscopic lymphadenectomy

## Abstract

**Background:**

This study aimed to propose a new surgical strategy, i.e., the transcervical video-assisted mediastinoscopic lymphadenectomy (VAMLA) with esophagectomy via the left transthoracic approach for patients with esophageal cancer (EC), and to compare the outcomes with those of esophagectomy via the right thoracic approach.

**Methods:**

From December 2014 to March 2016, 49 cases were enrolled in this non-randomized concurrent control study. Twenty-eight patients with EC who underwent transcervical VAMLA with esophagectomy via the left transthoracic approach were assigned into the study group, while 21 EC patients undergoing esophagectomy via the right transthoracic approach during the same period were enrolled into the control group. Operative outcomes including operative time, the numbers of removed lymph nodes, intraoperative blood loss, the length of hospital stay, and postoperative complications in both groups were evaluated and compared.

**Results:**

There were no significant differences in the baseline profiles between the two groups, and all patients in the two groups successfully underwent the surgery. There was a significant difference between transcervical VAMLA with esophagectomy via the left thoracic approach and esophagectomy via the right thoracic approach with regard to the number of all dissected lymph nodes [(29.0 ± 8.7) vs. (17.8 ± 8.1), *p* < 0.05], dissected superior mediastinal lymph nodes [(11.2 ± 5.0) vs. (3.7 ± 2.9), *p* < 0.05], and dissected in the recurrent laryngeal nerve lymph nodes [(5.6 ± 3.5) vs. (2.3 ± 2.1), *p* < 0.05]. No significant differences were observed in the operative time, intraoperative blood loss, length of postoperative hospital stay, number of dissected abdominal lymph nodes, postoperative pulmonary complications (pneumonia and atelectasis), anastomotic fistula, chylothorax, and vocal cord paralysis (*p* > 0.05).

**Conclusion:**

Transcervical VAMLA combined with esophagectomy via the left thoracic approach appears technically feasible and safe and shows advantages in the number of dissected superior mediastinal lymph nodes, suggesting that it may serve as a new treatment option for patients with esophageal carcinoma.

## Background

Esophageal cancer (EC) is one of the worst malignant digestive neoplasms and the sixth leading cause of cancer-related deaths around the world [1], producing 482,300 newly diagnosed cases worldwide in 2014 [2]. Because of the extensive network of esophageal lymphatics, early regional tumor advancement and metastasis to regional lymph nodes often occur, contributing to an 85% mortality rate [3]. Surgeries including transhiatal esophagectomy and transthoracic approaches remain the mainstay of treatment in achieving loco-regional control in EC patients and offer the best chance for cure in early localized and locally advanced diseases [4].

For the radical resection of EC, the most common open surgical protocols include transthoracic and transhiatal esophageal resections [5, 6]. The Ivor Lewis (laparotomy and right thoracotomy) and Sweet approaches (through a single left-sided thoracic incision) are two most widely used transthoracic esophagectomy techniques [5, 7, 8]. The traditional left transthoracic approach allows for less incision, less trauma, relatively small risk of perioperative complications, and good exposure of the middle-lower thoracic esophagus; however, it poses difficulties in mediastinal lymph node dissection, especially in bilateral recurrent laryngeal nerve (RLN) lymph node dissection [9]. In contrary, right thoracotomy combined with the transabdominal approach promises better mediastinal and abdominal lymph node dissection; however, it increases the number of required abdominal incisions, aggravates trauma in patients, and poses difficulties in bilateral RLN lymph node dissection which may damage the RLN, limiting its wide range of applications [10]. Additionally, the transcervical and transdiaphragmatic approaches without thoracotomy are limited to early-stage diseases and patients with high risks of surgery [11–13].

Recently, the widely used minimally invasive esophagectomy (MIE) for the patients with EC has reduced the surgical trauma and the incidence of complications; however, this operation still requires abdominal incisions that increase trauma and may cause complications such as abdominal incision infection and hernia [14]. Further, the application of MIE requires surgeons to learn the operation management techniques such as familiarizing laparo-thoracoscopy techniques, which may increase the difficulty of the surgery and the operating costs. Additionally, the greatest difficulty in MIE lies in the exposure of the bilateral RLN and the dissection of RLN lymph nodes dissection, which may damage the RLN and increase the operation difficulty [9].

The application of surgical resection with the left or right transthoracic approach in patients with EC is always controversial, and the core dispute focuses on which approach can achieve better mediastinal lymph node dissection [15]. The left thoracic approach is considered to be the main approach used in China; however, it is criticized for the limited extent of lymphadenectomy, especially in the upper mediastinum [15, 16]. Thus, finding an effective method for upper mediastinal lymph node dissection is imperative. Video-assisted mediastinoscopy can be a useful method to expose the superior mediastinal anatomic structure and remove the superior mediastinal lymph nodes [17]. In this study, we proposed a new surgical approach, i.e., the application of transcervical video-assisted mediastinoscopic lymphadenectomy (VAMLA) combined with the left transthoracic esophagectomy for patients with EC, and to compare its outcomes with those of esophagectomy via the right transthoracic approach. The study may offer a potential treatment option for patients with EC.

## Methods

### Patients

In this non-randomized concurrent control trial, we identified consecutive 49 patients with EC between December 2014 and March 2016. All patients were divided into either study group or control group by the method that was not random. The chief surgeon defined and managed the alternatives based on the wills of patients and their family members. In order to reduce the deviation, only the EC patients who met the criteria of VAMLA combined with esophagectomy via the left transthoracic approach were enrolled in the control group. Patients in the study group underwent VAMLA combined with esophagectomy via the left transthoracic approach, while others in the control group received esophagectomy via the right transthoracic approach. The surgery was performed by the same group of surgeons, who was well trained and had performed more than 200 cases of esophagectomy.

The inclusion criteria were as follows: (1) tumor located in the middle-lower thoracic region, a clear pathological diagnosis confirmed using gastroscopy and endoscopic ultrasound, clinical stage under cT3N1M0 by preoperative evaluation according to UICC 7th edition TNM staging [18], and no preoperative adjuvant radiochemotherapy; (2) ability of the lungs to tolerate lung ventilation thoracotomy with normal basic functions of the heart, liver, kidney and other major organs; and (3) no obvious enlarged mediastinal lymph node (> 1 cm) via imaging assessment or no significant enlarged cervical lymph nodes (> 1 cm) via neck computed tomography evaluation. The exclusion criteria were as follows: (1) clear contraindications to surgery; (2) history of malignant tumors, left chest surgeries, or severe left chest adhesions; and (3) not suitable for cervical mediastinoscopy due to serious carotid arteritis, large range of calcification of the aortic arch or innominate artery, and a huge goiter. The study was approved by the Ethical Committee of Hunan Provincial Tumor Hospital, Changsha, China. The informed consent was obtained from patients before the surgery.

### Surgery methods

After the complete surgical preparation, all patients underwent general anesthesia and double-lumen tracheal tube intubation.

**Fig. 1:**
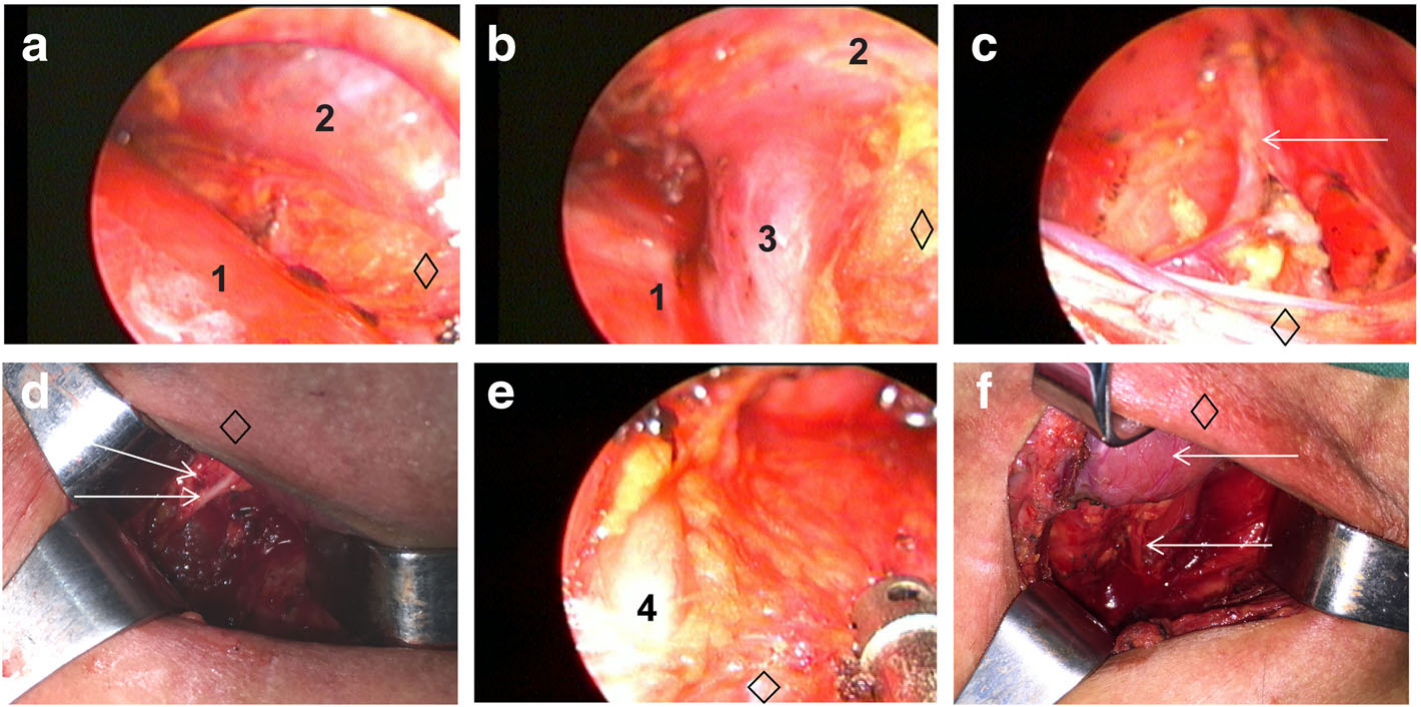
Anatomy under mediastinoscopy. **a** Sweeping the superior vena cava tracheal space between the lymph nodes. **b** Dissecting the azygos vein. **c** Dissecting the right lower laryngeal nerve; the arrow indicates the right recurrent laryngeal nerve in the chest. **d** Dissecting the right upper recurrent laryngeal nerve; the upper arrow indicates the thyroid on the right side, and the lower arrow indicates the right upper recurrent laryngeal nerve. **e** Anatomy of the left lower recurrent laryngeal nerve. **f** Anatomy of the left side of the right upper recurrent laryngeal nerve; the upper arrow indicates the thyroid on the left side, and the lower arrow indicates the left upper recurrent laryngeal nerve. 1 indicates the trachea, 2 indicates the precava, 3 indicates the azygos vein, and 4 indicates the left side of the recurrent laryngeal nerve in the chest. Diamond represents the cranial side

1. VAMLA combined with the left transthoracic esophagectomy was divided into two steps:
  First, the patients were placed in the right lateral decubitus position, and a routine left posterolateral thoracotomy was performed. The esophagus was then freed, and a gastric tube was created. Lymph nodes in the thoracic cavity and abdominal cavity including the nodes along the esophagus, the right and left cardiac node, the nodes along the left gastric artery, and greater and lesser gastric curvatures were resected. For three cases with lower thoracic tumors in this group, the esophagogastric anastomosis was constructed below the aortic arch. The remaining 25 cases underwent left neck anastomosis as follows: esophageal tumor was resected about 2 cm above the edge of the esophageal tumor, and the stump of the remnant esophagus was embedded using sterile finger cot, fixed with silk suture, and then hung with highest point of the gastric conduit and ligated with silk suture to prepare the neck incision, gastric tube traction, and esophagogastric anastomosis. Additionally, the gastric conduit should be placed carefully in the esophageal bed with a particular direction. Finally, the placement of the chest drainage tube and the suture of thoracic incision were performed after the diaphragm was closed.
  Second, the patients were repositioned in a supine position with shoulder padded high and head hypsokinesis as far as possible. We made an approximate 5-cm transverse incision 2 cm above the sternal notch. The skin, subcutaneous tissue, anterior muscles, and tracheal fascia were incised and separated with the finger along both sides of the trachea. Subsequently, the mediastinoscope was inserted along the right side of the trachea, and the lymph nodes in the space between the trachea and the superior vena cava were removed up to the bottom of the innominate artery and down to the bottom of the azygos vein (Fig. 1a, b). The innominate artery was risen properly to expose right RLN by the mediastinoscope and right RLN lymph nodes were dissected, and then RLN of the thoracic segment was separated and exposed under the mediastinoscope and the lymph nodes were resected (Fig. 1c). The right neck sheath and trachea were pulled with the retractor to both sides to expose the cervical segment of right RLN, and then the corresponding lymph nodes were removed. The protection of the parathyroid gland from injury is necessary during the procedure (Fig. 1d). The mediastinoscope can be used to expose the right RLN and to determine whether the RLN lymph nodes were completely dissected. Then, the mediastinoscope was inserted into the left side of the trachea and lower cervical part, and the upper thoracic part of the esophagus was separated and exposed to the free level of the thoracic cavity. The left RLN was isolated and exposed down to the left main trachea, followed by the dissection of left RLN lymph nodes (Fig. 1e). Then the left side of the neck sheath and trachea were pulled with the retractor to both sides to free the superior segment of the left laryngeal nerve up to the thyroid side, and the left upper RLN lymph nodes were removed (Fig. 1f). Likewise, the integrity of RLN lymph nodes dissection should be identified as well. In the parathyroid level, the stomach tube was pulled to the neck, with esophagogastric anastomosis done using line cutter or circular anastomat (Johnson & Johnson Co., USA). Indwelling gastrointestinal decompression tube and jejunum nutrition tube were routinely used. Further, the neck drainage tube was placed, and the neck incision was sutured.
2. Radical esophageal surgery via right transthoracic approach was performed with the similar steps reported in the previous literature [9, 14]. Intraoperative frozen margin sections were used, and all the margins had negative findings. Eight patients underwent chest anastomosis, and 11 patients underwent neck anastomosis.
3. After surgery, all cases would be nursed and treated in the same ICU.

### Data collection and follow-up

The primary endpoint of the study was the incidence of major complications in both groups. Major complications included anastomotic fistula, chylothorax, vocal cord paralysis, and respiratory complications (pneumonia and atelectasis). Four weeks after the operation was considered as the cut-off point for postoperative complication evaluation, while perioperative mortality was defined as death within 30 days. Secondary endpoints included the number of dissected regional lymph nodes, perioperative mortality, operative time, intraoperative blood loss, and the length of postoperative hospital stay. The mean follow-up duration in both groups was 12 months (range 6–20 months).

### Statistical analysis

The SPSS18.0 statistical software was used for the data analysis. The continuous data were expressed as mean ± standard deviations, and the Mann-Whitney *U* test [19] was used. The categorical variables were expressed as frequencies using the *χ*^2^ test, and the difference with *p* < 0.05 (bilateral) was considered statistically significant.

## Results

### Clinical data

**Fig. 2:**
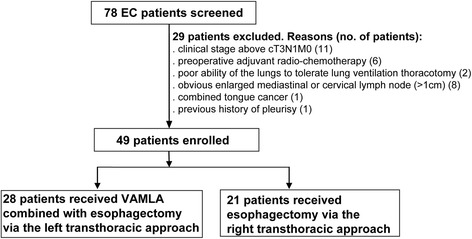
Flow chart of patient enrollment into the study

According to the abovementioned criteria, 28 patients (28 men, 24 with squamous cell carcinoma and 4 with sarcoma) with EC in the study group received VAMLA combined with esophagectomy via the left transthoracic approach, while the 21 patients (19 men and 2 women, 18 with squamous cell carcinoma and 1 with sarcoma) in the control group received esophagectomy via the right transthoracic approach (Fig. 2). According to the UICC 7th edition TNM staging [20], the stomach was used to reconstruct the esophagus. General information on these two groups of patients is in Table 1. Data indicated that the baseline characteristics between the two groups have no differences.

**Table 1:** Patient and tumor demographics in two groups

Patient demographics	Study group	Control group	*p* value
Gender			0.18
Male	28	19	
Female	0	2	
Age (years)	61.8 ± 5.1	60.1 ± 4.9	0.24
Comorbidity	5	6	0.37
Arrhythmia	1	2	
Hypertension	2	3	
Diabetes	1	1	
Location			0.13
Middle	13	14	
Lower	13	4	
Middle-lower	2	3	
Differentiation			0.22
G1	2	2	
G2	22	15	
G3	2	3	
Sarcoma	4	1	
Staging			0.53
I	6	2	
IIA	3	1	
IIB	7	10	
IIIA	7	5	
IIIB	4	3	
IIIC	1	0	

Study group—VAMLA + left transthoracic approach; control group—right thoracic approach

### Perioperative comparison and follow-up

One patient in the study group suffered from anastomotic fistula with severe pulmonary infection and then passed away even after intensive treatment. In the control group, anastomotic fistulas occurred in two cases and were cured after conservative treatment. There were no significant differences in operative time, intraoperative blood loss, postoperative hospital stay, postoperative respiratory tract complications (including pneumonia and atelectasis), anastomotic fistula, chylothorax, and vocal cord paralysis between the two groups (Table 2). No patients were treated with prolonged ventilatory support; instead, the patients were treated only with intensive anti-infection therapy, bronchoscopic suctioning, and pulverization inhalation to dispel phlegm.

**Table 2:** Comparison of perioperative data of two groups of patients

	Study group	Control group	*p* value
Operative time (min)	363.4 ± 78.8	349.6 ± 86.3	0.56
VMALA time (min)	80.6 ± 15.7	–	
Blood loss (ml)	321.0 ± 137.0	384.0 ± 181.1	0.15
Hospital stay (day)	12.0 ± 4.3	15.9 ± 20.3	0.39
Complications			
Respiratory complications	6	5	0.84
Anastomotic fistula	1	2	0.56
Chylothorax	1	0	1.0
Vocal cord paralysis	4	3	0.44

Study group—VAMLA + left transthoracic approach; control group—right thoracic approach

In the study group, one patient occurred to have multiple pulmonary and bone metastases 8 months after the operation, and two cases confronted anastomotic recurrence (13 and 15 months after the operation, respectively, including one case of only anastomotic recurrence and another case of anastomotic recurrence with mediastinal and abdominal lymph nodes recurrence). In the control group, one case had multiple pulmonary metastases 9 months after the operation, and three cases had anastomotic recurrences (9, 12, and 14 months, respectively), including two cases of mediastinal and abdominal lymph nodes recurrence.

### Lymph node dissection

In the study group, the number of total dissected lymph nodes, superior mediastinal lymph nodes, and RLN lymph nodes was 29.0 ± 8.7, 11.2 ± 5.0, and 5.6 ± 3.5, respectively; in the control group, the number was 17.8 ± 8.1, 3.7 ± 2.9, and 2.3 ± 2.1, respectively. The total number of dissected lymph nodes, the harvested number of superior mediastinal lymph nodes, and the number of RLN lymph nodes were significantly different between the two groups (*p* < 0.05, Table 3). The number of dissected abdominal lymph nodes in the study and control groups was 6.5 ± 3.3 and 5.5 ± 2.8, respectively. There was no significant difference concerning the number of dissected abdominal lymph nodes between both groups (*p* > 0.05).

**Table 3:** The comparison of lymph node dissection between the two groups

	Study group	Control group	*p* value
Total number	29.0 ± 8.7	17.8 ± 8.1	0.00
Superior mediastinum	11.2 ± 5.0	3.7 ± 2.9	0.00
Right RLN	3.0 ± 2.0	1.3 ± 1.2	0.00
Left RLN	2.6 ± 1.7	1.0 ± 1.0	0.00
Bilateral RLN	5.6 ± 3.5	2.3 ± 2.1	0.00
Enterocoelia	6.5 ± 3.3	5.5 ± 2.8	0.31
Total lymph node metastasis (%)	15 (53.6)	11 (52.4)	0.93
RLN LN metastasis (%)	7 (25)	2 (9.5)	0.16

Study group—VAMLA + left transthoracic approach; control group—right thoracic approach

*RLN* recurrent laryngeal nerve, *RLN LN* recurrent laryngeal nerve lymph node

## Discussion

Radical esophagectomy via either left or right transthoracic approach for patients with EC is still controversial, and which method is more efficient to achieve superior mediastinal lymph node dissection needs further discussion. Esophagectomy via the right transthoracic approach is considered as the standard surgical strategy for EC cases in western countries [21]. On the contrary, in China and some Asian countries where EC is with a high incidence, the left transthoracic approach is used as the main surgical approach in the current status quo.

A recent report showed that preoperative chemotherapy in patients with stage II/III esophageal squamous cell carcinoma contributed to better prognosis [22]. However, decisions regarding neoadjuvant therapy for esophageal cancer remain a subject of controversy [23]. Evidence suggests neoadjuvant therapy may lead to worse surgical results [24]. This study covered cases with no preoperative adjuvant radiochemotherapy. The patients with EC were enrolled from December 2014, and radical surgery was selected for these patients using preoperative evaluation. For new-enrolled EC cases in stage II/III in later study, we will recommend preoperative neoadjuvant therapy for them. On the other hand, in this study, patients with no obvious enlarged mediastinal or cervical lymph node were included. We recommended preoperative neoadjuvant therapy or neck lymph node dissection for those patients with obvious enlarged mediastinal or cervical lymph node because direct surgical treatment was not suitable for these cases. Moreover, although only patients without enlarged nodes were included, in fact, more than 50% of dissected nodes were pathologically positive in this study. Actually, the accuracy of CT for correct assessment of lymph node metastasis is reported only about 40%, and histopathological examination of lymph node metastasis serves as the reference standard [25]. Most cases in our study were in T2 or T3 stage. Thus, it is possible that lymph nodes were characterized as negative during preoperative evaluation while the incidence of positive lymph nodes after surgery was higher.

Lymph node dissection plays an essential role in EC radical resection [26, 27]. Lymph node metastasis ratios and lymph node metastasis numbers are independent risk factors for EC prognosis [28, 29]. The RLN lymph node is one of the most common metastatic and relapsing sites in EC even after radical resection, and the rates of this metastasis in upper thoracic EC are as high as 43.3% [30, 31]. The RLN lymph node metastases are considered as independent predictors of cervical lymph node metastases [32, 33]. Evidence showed that the 3-year survival rate was 29.3% in patients with RLN lymph node metastases after surgery, while 58.2% in patients without RLN lymph node metastases (*p* < 0.05) [34]. Thus, the RLN lymph node dissection is considered beneficial for EC patients and is the key point of lymph node dissection for EC treatment [21, 34, 35]. However, the dissection of RLN lymph nodes is the main difficulty in EC radical surgery. Since RLN travels longer with various anatomical positions, it is pretty vulnerable during operation, and the damage will lead to hoarseness, cough, aspiration pneumonia, pulmonary infection, respiratory failure, and even death [32]. Additionally, as bilateral RLN impaired, serious complications may occur such as life-long tracheostomy.

In the study of Matsuda et al., the total number of dissected LNs was 20.02 ± 8.16 and 27.93 ± 11.75, respectively, in the thoracic duct (TD)-preserved and TD-resected groups via right transthoracic approach [36], which showed a little more number of dissected LNs compared with that of the control group of this study (the number of total dissected lymph nodes is 17.8 ± 8.1). However, our highly skilled surgeons had many years of experience performing surgical resection of EC. Our hospital is a professional clinical diagnosis and treatment center of esophageal cancer in China, and there is strict quality control of radical esophagectomy and lymph node dissection. Moreover, it met the National Comprehensive Cancer Network (NCCN) guidelines for esophageal cancer which indicate at least 12 lymph nodes should be removed [37]. Although the procedure of bilateral RLN lymph node dissection via left transthoracic approach has been reported in some study [38], the method is tough to perform and is not conducive to the widespread application. Therefore, the development of an efficient method of upper mediastinal lymph node dissection for EC radical surgery via left transthoracic approach is undoubtedly an excellent complement and optimization. The video-assisted mediastinoscopy has advantages in good exposure on the upper mediastinal anatomic structure and the dissection of the upper mediastinal lymph nodes, especially in bilateral RLN lymph node dissection [13, 39]. Thus, the video-assisted mediastinoscopy may be a potential complement for EC radical surgery via left transthoracic approach, promoting the application of the method for EC radical surgery.

Transcervical mediastinoscopy is a relatively mature surgical procedure in thoracic surgery, which is mainly used for the diagnosis of a mediastinal mass, lymph node biopsy, and preoperative staging of lung cancer. In 1990, Buess and Becker [39] first reported the video-assisted mediastinoscopy as a treatment for EC. According to the present research around the world, video-assisted mediastinoscopy for EC treatment is based on transcervical combined with the transhiatal operation to isolate the cervical and upper thoracic esophagus and to remove the mediastinal lymph nodes [40, 41]. However, there is no report about the use of transcervical VAMLA auxiliary for the radical operation of EC via left transthoracic approach.

VAMLA combined with radical operation via left transthoracic approach for EC has the following potential advantages: (1) not only retaining the benefits of the traditional esophagectomy via the left transthoracic approach but also achieving the superior mediastinal lymph nodes and bilateral RLN lymph node dissection without increasing surgical incisions; (2) satisfactory exposure of the upper mediastinal anatomical structure, in particular, the exposure of bilateral RLN ,and better lymph node dissection; (3) achieving the cervical and upper thoracic esophageal dissociation simultaneously; and (4) less trauma and bleeding, as well as less postoperative pain and complications.

Our results indicated that the total number of dissected lymph nodes and the numbers of upper mediastinal lymph nodes and RLN lymph nodes were significantly higher in the study group than that in the control group, while the number of dissected abdominal lymph nodes was similar in both groups. It suggested that VAMLA combined with radical operation via left transthoracic approach for EC was not inferior to esophagectomy via the left transthoracic approach in the dissection of abdominal lymph nodes. Furthermore, it has distinct advantages in dissecting the upper mediastinal lymph nodes, especially for the RLN lymph node. In this study, the RLN lymph node metastatic rate was 25% (7/28), namely four cases of right RLN lymph node metastasis and three cases of left RLN lymph node metastasis, which was similar with that reported in domestic and foreign relevant literature [13, 34].

There were no significant differences in postoperative complications including respiratory system complications, arrhythmia, chylothorax, anastomotic fistula, postoperative hospital stay, and vocal cord paralysis between the two groups. Four patients (14%, 4/28) in the study group were subjected to RLN paralysis, and the symptom in three of them significantly alleviated after 3 months of operation. The incidence of nerve palsy, however, has been reported to be 3.1–22.5% in other studies [42]. The possible reason is that the right transthoracic approach is not sufficient for the RLN isolation which limits the resection of lymph nodes, while VAMLA did not increase the risk of RLN damage due to its profits in the exposure of this area although the uncovered time of RLN lasts the entire operation. A study also showed that intraoperative application of single-chamber tracheal tube EMG signal and EZ to monitor the RLN could reduce the frequency of RLN injury [43]. Meanwhile, it also should be noticed that the mediastinoscopy operation by itself causes the incidence of complications approximately 0.5%, including injury of superior vena cava, azygos vein, innominate artery, and other large vascular damage (mediastinal infection, tracheoesophageal injury, and misdiagnosed parathyroid risk) [44]. Nevertheless, as long as the surgeons are meticulous and well trained, VAMLA is a very safe operation.

In the present study, VAMLA was used to make up the deficiency of the dissection of recurrent laryngeal nerve lymph nodes (RLN LNs) in esophagectomy via left transthoracic approach. Although the Ivor Lewis approach is the most routinely performed approach by most surgeons [45], we also need to perform esophagectomy via left transthoracic approach on some patients in clinical, such as the patients with right-sided chest empyema, right chest surgeries, or severe right chest adhesions and patients with combined pulmonary nodules at the left lung that need to be removed and determine pathology. VAMLA with esophagectomy via the left transthoracic approach undoubtedly shows its advantages and importance. So, in the development of this new strategy, we have made a few improvements, mainly in the exposure procedures of RLN. As a new protocol, VAMLA with esophagectomy via left transthoracic approach possesses good learnability and popularization, although it requires a period of mediastinoscope learning curve and the related experience of esophagectomy. Apart from the experience of surgeons, the selection of EC patients to receive this novel operation bases on their clinical stage, and all the patients under cT3N1M0 will be suitable for this protocol. However, VAMLA combined with esophagectomy via left transthoracic approach still has some shortcomings, such as positional change during surgery, the requirement of video-assisted mediastinoscopy instruments, and skilled surgeons. Besides, more meticulous operating are also needed because the manipulating space is limited, and fractional resection of large lymph nodes is sometimes needed. Moreover, a limitation of this study was the relatively short period of follow-up, and the efficacy of this surgery on the prognosis in EC patients needs further follow-up study. Finally, it has been reported that 38.3–56.5% of the RLN LN metastases accompanied by the supraclavicular lymph node metastasis, which means RLN LN should be considered an important indication for supraclavicular lymph node dissection [32, 38]. However, the group of positive RLN LNs cases in this study did not undergo the cervical lymph node dissection, and the significance needs further investigations.

## Conclusions

In summary, VAMLA combined with esophagectomy via left transthoracic approach is technically feasible and safe. It shows advantages in the number of the upper mediastinal lymph node dissection, especially the RLN lymph node dissection, which suggests that it may offer a new treatment option and a complementary therapy for patients with EC. However, more cases and long-term follow-up should be included in the further study.
